# Response to: Comment on “Intervention Effects of a School-Based Health Promotion Programme on Obesity Related Behavioural Outcomes”

**DOI:** 10.1155/2015/347590

**Published:** 2015-08-09

**Authors:** Susanne Kobel, Tamara Wirt, Anja Schreiber, Dorothea Kesztyüs, Sarah Kettner, Nanette Erkelenz, Olivia Wartha, Jürgen M. Steinacker

**Affiliations:** Division of Sports and Rehabilitation, Department of Internal Medicine II, Ulm University Medical Centre, Frauensteige 6, Haus 58/33, 89075 Ulm, Germany

Evaluating multicomponent health programmes in public institutions is always a complex task and requires careful planning [1]. Reporting the outcomes in a precise and understandable manner is a further challenge for the researcher [2].

The points raised by Li et al. [3] are very valid and were also considered by us when writing the paper. Although outlined by Dreyhaupt et al. [4], not all planned analyses were incorporated in our paper [5]. Since the paper [5] (especially Methods) ended up being considerably substantial, we refrained from adding results of further statistical analyses to the paper. Further, in previous paper concerning cross-sectional results of this study, we referred to the respective consideration of clustering effects including adequate statistical methods [6, 7]. Since almost no clustering effects were observed, we refrained from introducing them in the present paper, even due to the associated complexity [2].

However, we have compared the results of the logistic regression models used and published in the paper with the results of generalised linear mixed models considering possible clustering effects in schools. Only a slight difference between odds ratios (ORs) was derived from the logistic regression models and the ORs from generalised linear mixed models for the variables “soft drink consumption” and “skipping breakfast.” Hence, we refrained from explaining and mentioning it in the paper.

To reveal these above-mentioned slight differences between the results published in the paper and those derived from mixed models accounting for clustering, we included Table 1, which shows the results from both types of analysis. The ORs for our outcome variables physical activity, screen media use, soft drink consumption, and breakfast habits show no significant differences, and therefore we assume no clustering effects in our data.

## Figures and Tables

**Table 1:** Comparison of the results derived from logistic regression models and generalised linear mixed models.

	*n*	OR	95% CI
*Physical activity, MVPA on ≥4 days/week ≥60 minutes *	1386		
Logistic regression model		1.18	[0.92, 1.52]
Generalised linear mixed model		1.18	[0.92, 1.52]
*Screen media use, screen media ≥1 h/day *	1471		
Logistic regression model		0.75	[0.53, 1.06]
Generalised linear mixed model		0.75	[0.53, 1.06]
*Soft drink consumption, soft drinks ≥1 time/week *	1475		
Logistic regression model		0.96	[0.72, 1.28]
Generalised linear mixed model		0.93	[0.68, 1.29]
*Breakfast habits, skipping breakfast *	1480		
Logistic regression model		0.86	[0.58, 1.29]
Generalised linear mixed model		0.89	[0.57, 1.39]

OR: odds ratio; CI: confidence interval.
